# The clinical significance of heterozygous E148Q variant in patients with familial Mediterranean fever

**DOI:** 10.1007/s10067-026-08142-7

**Published:** 2026-05-08

**Authors:** Abdülvahhap Aktaş, Yousef Aljaber, Huda Avad, Döndü Ü. Cansu, Cengiz Bal, Cengiz Korkmaz

**Affiliations:** 1 Ministry of Health, Kutahya Evliya Celebi Training and Research Hospital, Kütahya, Türkiye; 2 School of Medicine, Eskişehir Osmangazi University, Eskişehir, Türkiye; 3 Department of Internal Medicine, Division of Rheumatology, Eskişehir Osmangazi University, Eskişehir, Türkiye; 4 Department of Medical Biostatistic, Eskişehir Osmangazi University, Eskişehir, Türkiye

**Keywords:** Familial mediterranean fever, Genotypes, Heterozygous E148Q, M694V, MEFV mutations

## Abstract

**Objectıve:**

Our objective was to compare the clinical characteristics of heterozygous E148Q-positive familial Mediterranean fever (FMF) patients with those of E148Q/M694, M694V-homozygous, and M694V-heterozygous-positive patients.

**Methods:**

Tel-Hashomer classification criteria were used to diagnose FMF. Exons 2, 3, 5, and 10 MEFV mutations were evaluated using the multiplex-PCR reverse hybridization method. The Tel-Hashomer FMF severity score was taken into consideration for FMF severity. The severity score was determined taking into account the period before using colchicine. The clinical features of FMF patients with the E148Q variant were compared to those with heterozygous E148Q plus M694V, and to patients with heterozygous or homozygous M694V mutations.

**Results:**

The study included 148 patients with FMF. E148Q heterozygosity was found in 14 patients (9.4%), M694V/E148Q positivity in 13 patients (8.7%), M694V heterozygosity in 49 patients (33.1%), and M694V homozygosity in 72 patients (49.6%). The disease began at an earlier age in those with M694V homozygosity compared to those with M694V heterozygosity and those with E148Q heterozygosity. However, there was no difference in disease onset age between those with M694V homozygous mutations and those with M694V/E148Q. As expected, disease severity scores, erysipelas-like erythema, and relative marriage rates were higher in those who were M694V homozygous. There was no difference between the groups in terms of fever, abdominal pain, arthritis/arthralgia, vasculitis, familial history, or frequency of ankylosing spondylitis.

**Conclusıon:**

Patients with heterozygous E148Q variant may exhibit main clinical features of FMF disease.

**Table:** 

**Key Points** • *About 10% of FMF patients have heterozygot E148Q variant*. • *The clinical characteristics of patients with E148Q variant may be similar to those of patients with Exon 10 mutation*.

## Introduction

Familial Mediterranean fever (FMF) is a monogenic autoinflammatory disease with the potential to cause secondary amyloidosis, accompanied by recurrent fever and serositis findings [1]. The diagnosis of FMF relies on clinical signs and is supported by genetic testing [2]. It is known that Mediterranean fever (MEFV) mutations on the short arm of chromosome 16, especially exon 10 mutations, are associated with disease pathogenesis. The most common mutations are M694V, M694I, V726A, and M680I mutations. The pyrin mutation causes the activation of the pyrin inflammasome, which results in increased production of interleukine-1β [3]. The debate on whether E148Q, as an exon 2 mutation, is a non-significant variant or a mutation that causes disease is still ongoing [4, 5]. The source of this debate stems from conflicting observations in patient cohorts and healthy controls. The fact that it is seen at a high frequency in healthy individuals leads to the idea that the variant is a benign polymorphism [4, 6]. However, an alternative and increasingly supported perspective advocates the pathogenic role of E148Q. The data supporting this view is that there are symptomatic patients with the E148Q variant, and they respond positively to colchicine treatment. Studies of patients with homozygous E148Q variants consistently show that most of these individuals are symptomatic and exhibit a clinical phenotype consistent with FMF. In addition, these patients often need and respond to treatment of colchicine [7]. While controversial, it is suggested that the E148Q variant may lead to spontaneous inflammasome activation, resulting in mild-to-moderate severity in FMF clinics [8].

The heterozygous state of the E148Q variant combined with exon 10 mutations can modify these mutations [9]. Most of the studies on E148Q are based on homozygous E148Q or E148Q with 10 exon mutations [7, 10], and most of them have been performed in pediatric-age patients [10, 11]. There has been no study designed like this in adult FMF patients. In this study, we evaluated the frequency of E148Q heterozygous positive patients and compared their clinical characteristics with those of heterozygous E148Q plus M694V and patients with heterozygous M694V or homozygous M694V mutations in adult FMF patients.

## Materials and methods

This study data was obtained from the data of our previous study, which examined the effect of FMF genotypes on disease severity and concomitant diseases [12]. While our previous study provided a broader mutational spectrum, the present study specifically focuses on the clinical impact of E148Q heterozygosity in comparison to M694V genotypes. We excluded other genotypes (such as E148Q homozygosity or compound heterozygosity with other mutations) to ensure a more homogenous study group and to realize the purpose of the work. In this study, FMF patient files were retrospectively examined. Tel-Hashomer classification criteria were used to diagnose FMF [13]. The Tel-Hashomer FMF severity score was taken into consideration for FMF severity [2]. The severity score was determined taking into account the period before using colchicine. In our present research, the patients were divided into four groups according to the mutations. Group 1 included the patients who had M694V/-, group 2 consisted of M694V/E148Q, group 3 comprised only E148Q, and group 4 included M694/M694V. The clinical characteristics of FMF patients with the positive E148Q variant were compared with those of other groups. The subjects’ written consents were obtained according to the Declaration of Helsinki, and the study was approved by the ethics committee of Eskişehir Osmangazi University.

### Mutation analysis

DNA was extracted from peripheral blood leukocytes using standart protocols (Invisorb Spin Blood Kit, STRATEC Molecular DmbH, D-13125;Berlin, Germany). Molecular analyses were performed within the framework of routine genetic testing. The presence of MEFV mutation was investigated in exons 2,3 5, and 10 by the multiplex-PCR reverse hybridisation method.

### Statistical analysis

We used IBM SPSS for Windows 21.0 to analyze the data. According to power analysis: There are 4 groups in the study. Group averages for age of onset were found to be 12 ± 11, 10 ± 9, 13.5 ± 12, 7 ± 6.1 with 80% power and a total of 140 patients were eligible to be included in the study. The Kolmogorov Smirnov test was used to determine the suitability of the variables for the normal distribution. We applied non-parametric tests to evaluate intergroup differences in age, age at disease onset, age at diagnosis, diagnostic delay, and severity scores. As a post-hoc analysis, the Bonferroni-corrected Dunn test is used to compare specific pairs of groups following a significant Kruskal–Wallis test. The analysis of the generated cross-tables was performed using chi-square tests (Pearson and Pearson’s exact chi-square). In summarizing the data, count (%), mean ± SD or Median (Q1; Q3) statistics were used for qualitative data. In all analyses, P < 0.05 is considered statistically significant.

## Results

### The characteristics of the patients

This study included 148 patients (64 male, 43.2%; 84 female, 56.8%). The average age was 33.4 ± 12.1 years. M694V heterozygosity was positive in 49 patients (33.1%) (Group 1), M694V/E148Q was positive in 13 patients (8.7%) (Group 2), E148Q heterozygote was found in 14 patients (9.4%) (Group 3), M694V homozygosity was positive in 72 patients (48.6%) (Group 4).

### Comparison of clinical features in groups (Table 1)

**Table 1:** Comparison of clinical characteristics of patients with E148Q and other mutations in patients with familial Mediterranean fever

		Group 1 M694V/— n = 49 Mean ± SD (Median Q1-Q3)	Group 2 M694V/E148Q n = 13 Mean ± SD (Median Q1-Q3)	Group 3 E148Q/— n = 14 Mean ± SD (Median Q1-Q3)	Group 4 M694V/M694V n = 72 Mean ± SD (Median Q1-Q3)	Differences (p)
**Age (year)**		31 ± 12.8 (31 (24;45)	31 ± 17 31 (19.5;45.5)	32 ± 12.3 32.5 (24.75;49.25)	31 ± 10.5 31(24;38;75)	0.8
**Age of onset (year)**		12 ± 11 12 (6;21)	10 ± 9 10(3.5;22)	13.5 ± 12 13.5 (7.5;24,25)	**7 ± 6.1** ^**a b**^ 7 (2;10.75)	**0.001** **0.043**
**Age of diagnosis (year)**		25 ± 11 25 (19;33)	30 ± 14 30(17.5;39)	26.5 ± 11 26.5(20.5;36.25)	20 ± 12 20(15;30)	0.06
**Delayed diagnosis (year)**		11 ± 12 11 (2;20)	14 ± 12 14(7;24)	12.5 ± 12 12.5 (2.75;23)	14 ± 11 14 (7.5–23.75)	0.42
**Severity score**		8 ± 2.6 8 (6;10)	7 ± 2.5 7 (5.5;8.5)	8 ± 2.7 8 (6;10.25)	**11 ± 2.6** ^**c**^ 11(9;12)	**0.0001** **0.001** **0.018**
**Abdominal pain (n, %)**		45 (91.8)	13 (100)	13 (92.8)	69 (95.8)	0.6
**Fever (n, %)**		47 (95.9)	12 (92.3)	13 (92.8)	67 (93)	0.9
**Arthritis (n, %)**		36 (73.4)	9 (69.2)	11 (78.5)	62 (86.1)	0.2
**Pleurisy(n, %)**		26 (53)	8 (61.5)	7 (50)	45 (62.5)	0.6
**Erysipel-like Erythema (n, %)**		20 (40.8)	3 (23)	7 (50)	**58 (80.5)** ^**d**^	**0.0001**
**Arthralgia (n, %)**		39 (79.5)	8 (61.5)	13 (92.8)	58 (80.5)	0.2
**Concomitant diseases**		16 (32.6)	0	2 (14.3)	15 (26.4)	0.08
**Family history (n, %)**		23 (53.2)	7 (53.8)	7 (50)	40 (56)	0.9
**Amyloidosis (n, %)**		1 (2)	0	1 (7)	11 (15)	0.052
**Vasculitis (n, %)**		4 (8.2)	0	2 (14.2)	2 (2.7)	0.169
**Appendectomy (n, %)**		9 (18.3)	4 (30.7)	4 (28.5)	27 (37.5)	0.159
**Ankylosing Spondylitis (n, %)**		8 (16.3)	0	0	10 (13.8)	0.189
**Consanguineous marriage (n, %)**		0	0	1	**9 (12.5)** ^**e**^	**0.03**
**The frequency of attacks before colchicine**	** < 1/month**	14 (28.5)	3 (23)	4 (28.5)	12 (16.6)	0.22
	**1–2/month**	17 (34.6)	7 (53.8)	1 (7.1)	24 (33.3)	0.07
	** > 2/month**	18 (36.7)	3 (23)	**9 (64.2)** ^**f**^	36 (50)	**0.09**

a: Grup 4 vs Grup 1

b: Grup 4 vs Grup 3

c: Grup 4 vs Grup 1; Grup 4 vs Grup 2; Grup 4 vs Grup 3

d: Grup 4 vs Grup 1, 2, 3

e: Grup 4 vs Grup 1, 2, 3

f: Grup 3 vs Grup 1, 2,4

The disease began at an earlier age in those with M694V homozygous, compared to those with M694V heterozygous and those with E148Q heterozygous. There was no difference in age of onset between patients with M694V homozygous and M694V/E148Q positive. As expected, disease severity scores, erysipelas-like erythema (ELE), and relative marriage rates were higher in those with M694V homozygous. There was no difference between the groups in terms of fever, abdominal pain, arthritis/arthralgia, vasculitis, familial history, frequency of ankylosing spondylitis, or frequency of pre-colchicine attacks. Amyloidosis was found in 11 (15%) patients with M694V homozygous, 1 patient with M694V heterozygous, and 1 patient with E148Q heterozygous (2% and 7%, respectively). Compared to other groups, although not as statistically significant, E148Q positive patients experienced more attacks prior to starting colchicine. Clinical characteristics of the groups are shown in Table 1.

## Discussion

The question of whether the E148Q variant causes FMF attacks has been investigated in most patients who have homozygous E148Q or it combined with other mutations [4, 5, 9, 11]. According to the results of these studies, homozygous E148Q positivity is associated with FMF clinical manifestations appearing at a later age, and the severity of the disease is less severe. In addition, when E148Q is combined with exon 10 mutations, the disease severity is similar to exon 10 mutations [7, 9].

We examined the clinical characteristics of FMF patients who were heterozygous for the E148Q variant and then compared them to those with the M694V mutation (exon 10 mutation). Our study is one of the first studies conducted in adult FMF patients investigating clinical features of E148Q heterozygous positive patients.

In Turkey, the frequency of E148Q positivity in FMF patients was reported to be different based on regions [14]. According to Ece et al., E148Q was the most prevalent mutation in the Southeast region [15], but it was the second most common mutation in the Eastern region [16–18], Northeastern region [19], and Central Anatolia [20]. As one of the central Anatolian region’s data, E148Q heterozygous positivity was found to be 9.4% in our study. A study in the Mediterranean region of Turkey revealed that R202Q was the most frequent mutation in individuals with an FMF pre-diagnosis, while E148Q was the second most frequent mutation [21]. Many ethnic groups live in Anatolia and more ethnic origin-based studies are needed to determine the real effect of these mutations on disease manifestations.

In terms of clinical features, E148Q heterozygous positive patients did not show a different clinical course than patients with mutations apart from M694V homozygous patients. It was noteworthy that amyloidosis manifested in one case with E148Q. As is expected, M694V-positive patients had severe severity scores, early age onset, and increased frequency of ELE.

The term "benign polymorphism" for E148Q is linked to its increased prevalence among healthy individuals. The carrier frequencies of the E148Q have been reported to be up to 53% in some Jewish populations [22], 12% in Turks, and 29% in various other populations [23, 24]. Tchernitchko et al. reported that 3.62% of FMF patients exhibited E148Q positivity, whereas their disease-free relatives demonstrated a 3.75% prevalence of E148Q. The prevalence of 694 V/E148Q compound heterozygosity was comparable between affected patients and their healthy relatives (3.94% and 4.2%, respectively). Consequently, the authors have interpreted the E148Q variation as a benign polymorphism [4].

Although the E148Q variant is regarded as a low-penetrance allele that is associated with a milder disease course and a potential modifier effect, our study showed that apart from patients homozygous for M694V, E148Q heterozygous patients did not exhibit a distinct clinical trajectory compared to other patient groups in terms of clinical manifestations. Notably, secondary amyloidosis was observed in 7% of cases. Our study showed that apart from patients homozygous for M694V, E148Q heterozygous patients did not exhibit a distinct clinical trajectory compared to other patient groups in terms of clinical manifestations. Notably, secondary amyloidosis was observed in 7% of cases. Furthermore, patients who are homozygous for M694V have an earlier disease onset compared to those who are heterozygous for M694V, but the onset age does not differ from that of patients positive for M694V/E148Q, suggesting that the E148Q variant may modify the influence of exon 10 mutations on the age of disease onset [7].

Aydin et al. compared patients with a positive E148Q mutation (heterozygote and homozygote) with those with M694V homozygous and patients with compound heterozygous E148Q with 10 exons. They found that those with the E148Q variant had a typical FMF attack, but the disease appeared and progressed more slowly at a later age [10]. According to Topaloglu et al., E148Q homozygous positive individuals had a late onset of disease and experienced a moderate or severe course of illness [25].

Gershoni-Baruch et al. demonstrated that homozygotes for the M694V mutation and the complex V726A/E148Q allele exhibit the most severe manifestations and frequently experience renal amyloidosis [26]. Authors demonstrated that the morbidity associated with the complex V726A/E148Q allele significantly exceeds that associated with the V726A allele, providing evidence that the E148Q mutation is not a benign polymorphism.

In 169 FMF patients with secondary amyloidosis, 11.2% showed E148Q/E148Q positivity, while the M694V homozygous ratio was 60.6% and the M694V/E148Q ratio was 14.8%. Researchers have suggested follow-up of the homozygous E148Q genotype for amyloidosis [27].

Tirosh and colleagues evaluated 21 E148Q heterozygous positives who were sent with an FMF prediagnosis. Of these, only 6 patients met the Tel-Hashomer criteria; only 1 showed the necessity of colchicine. As a result, the authors suggested that in their cohort, E148Q heterozygosity would not be considered a disease-causing variant [11]. However, the average patient age in this study is 6.9 years. The symptoms of FMF associated with E148Q mutations manifest at a later age, and individuals with E148Q in the pediatric population are less likely to fulfill the FMF diagnostic criteria; additionally, the mild progression of the disease in these patients may result in a missed diagnosis. The mean age of our patient cohort was 31.4 years. The average age of disease onset of our patients was 13.5 ± 12 years.

We do not know exactly why the disease appears as age increases. As time goes on, greater exposure to environmental factors that trigger attacks can be a cause. For example, the most important trigger for FMF attacks is stress [28]. In the adult age group, stress may be more important than in the childhood age group, as adults often face more significant stressors related to work, family responsibilities, and financial pressures that can exacerbate FMF attacks.

Ece et al. found that the clinical features of all E148Q-positive (homozygote plus heterozygote) patients had shorter attack times, fewer severity scores, and less ELE frequency than those of the M694V mutation [15]. Fujimoto et al. showed that the FMF course was more severe in those with E148Q-compound heterozygous than in those with E148Q heterozygous Japanese FMF patients. According to this study, abdominal pain and arthralgia were more common [29]. The E148Q variant was considered a mild mutation by Eyal et al., but they asserted that when it is coupled with other pathogenic variants, it can become a functional mutation [30].

The small number of patients in this study limits its scope, and it makes it difficult to make strong conclusions. Another limitation relates to the frequency of attacks reported by the patients. The frequency of pre-colchicine attacks was greater numerically in E148Q-positive patients, though not statistically significant. This result seems like a contradictory outcome. This discrepancy may be related to patients misremembering or exaggerating the frequency of attacks. Patients may have reported subjective symptoms due to factors unrelated to FMF attacks. Such discrepancies may arise from patients misunderstanding the definition of "attack" or from physicians inadequately explaining the definition to patients. Therefore, it is important to conduct such studies with a larger number of patients and in conditions where the definition of classic attacks is more clearly explained to patients.

Based on our previous cohort data [12], other genotypes, including E148Q homozygosity and compound heterozygosity with other mutations (e.g., M694V/E148Q/R202Q; M694V/E148Q/V726A; M680I/E148Q; and 148Q/P369S/M694V/E148Q), were identified in a very limited number of cases. To maintain the primary focus on the clinical significance of isolated E148Q heterozygosity, these rare complex alleles were not included in the current analysis.

As a result, we found that the E148Q heterozygous positive patients' rate accounts for 9.4% of patients and shows typical FMF characteristics. The higher E148Q positivity rate in different regions of Turkey suggests that ethnic and environmental factors may be important in the phenotype of the disease.

It has been reported that E148Q-positive patients have an atypical course of the disease and delayed diagnosis [31]. On the other hand, E148Q-positive patients can also develop amyloidosis, despite its rarity [32]. Therefore, clinicians should pay attention to the treatment of such patients with colchicine, as it has been shown to help manage symptoms and improve outcomes in these cases.

## Conclusion

Although similar studies in the pediatric age group exist in the literature, it will be necessary to conduct such studies in different ethnic groups and adult FMF patients because the E148Q variant may exhibit different phenotypic characteristics depending on environmental factors as well as ethnicity. These research studies with a significantly larger patient population will provide additional information regarding the E148Q variation.
